# Occurrence of Methicillin-Resistant *Staphylococcus* spp. on Brazilian Dairy Farms that Produce Unpasteurized Cheese

**DOI:** 10.3390/toxins12120779

**Published:** 2020-12-08

**Authors:** Laryssa Freitas Ribeiro, Rafael Akira Sato, Andressa de Souza Pollo, Gabriel Augusto Marques Rossi, Luiz Augusto do Amaral

**Affiliations:** Faculdade de Ciências Agrárias e Veterinárias, UNESP—Universidade Estadual Paulista, Via de Acesso Paulo Castellane, s/n, Jaboticabal CEP 14884-900, Brazil; jaspionvet04@gmail.com (R.A.S.); andressa_unesp@yahoo.com.br (A.d.S.P.); gabrielrossiveterinario@hotmail.com (G.A.M.R.); lamaral@fcav.unesp.br (L.A.d.A.)

**Keywords:** antimicrobials, MRS, RAPD, public health, resistance

## Abstract

Methicillin-resistant *Staphylococcus* spp. (MRS) have been identified in several foods, including dairy products. Studies are needed about their occurrence and genetic diversity in the dairy production chain in order to gain a better understanding of their epidemiology and control. This study therefore focuses on isolating and characterizing MRS strains detected in milk used in the production of Brazilian artisanal unpasteurized cheeses. To this end, samples were collected from bovine feces, the hands of milkmen, milking buckets, sieves, unpasteurized milk, whey, water, artisanal unpasteurized cheeses, cheese processing surfaces, cheese handlers, cheese trays, cheese molds, and skimmers at five dairy farms located in the state of São Paulo, Brazil. Colonies suggestive of *Staphylococcus* spp. were subjected to multiplex PCR to confirm the presence of *Staphylococcus aureus* and to detect the *mecA* gene. Sixteen isolates containing *mecA* gene were detected in samples from unpasteurized cheese and from cheese handlers. None of these isolates were positive to enterotoxin genes. These 16 isolates were subjected to antimicrobial susceptibility tests, which revealed they were resistant to oxacillin, penicillin, and cefepime. Using gene sequencing, the MRS isolates were identified as *S. haemolyticus, S. hominis*, and *S. epidermidis*. Furthermore, isolates from cheese handlers’ hands and artisanal unpasteurized cheese presented high genetic similarity by random amplified polymorphic DNA (RAPD-PCR) analysis, which indicates cross contamination during cheese production. Thus, we found that people directly involved in milking and cheese processing activities at small dairy farms are a potential source of contamination of MRS strains in unpasteurized milk and cheese, representing a risk to public health.

## 1. Introduction

The uncontrolled use of methicillin led to the emergence of methicillin-resistant *Staphylococcus* spp. (MRS), which poses a public health risk. The most widely reported MRS species is *Staphylococcus aureus* (MRSA). However, MRS strains have also been reported in veterinary medicine [1], and animal to human transmission has been described [2], underscoring the need to monitor these microorganisms and their susceptibility to antimicrobials in order to reduce their risk to public health. MRS strains are of great importance in public health owing to their opportunistic ability as a cause of mastitis, source of zoonotic infection, and reservoir of antimicrobial resistance genes in dairy farms [3]. Even though the coagulase-positive species (*S. aureus*) is more pathogenic and directly associated with severe mastitis, coagulase-negative staphylococci (CoNS) have been increasingly recognized as a cause of clinical and subclinical mastitis [4]. It occurs because CoNS are highly frequently detected in raw milk samples. They are widespread in dairy farms environment and the occurrence of methicillin resistance in these species is frequently higher than in *S. aureus* [5].

The first researchers to report a fatal MRSA foodborne outbreak were [6], and since then numerous microbiologists have evaluated the occurrence of methicillin-resistant strains in food and animals [7,8]. The importance of methicillin-resistance in farming and food production stems from the possibility of zoonotic infection of consumers and workers involved in animal husbandry [9]. Regarding food safety, MRSA have been identified in samples from food handlers in Brazil [10] and in milk and dairy products in several studies worldwide. In Mexico, MRSA was detected in 18.1% of artisanal cheese samples [11] and in Turkey, 30% of milk samples, 18% of clotted cream, and 34% of cheese samples were contaminated with MRSA [12]. Enterotoxigenic MRSA under favorable conditions can cause staphylococcal food poisoning owing to the production of enterotoxins in foods, nonetheless the severity of infection is not related to the antimicrobial resistance profile and outbreaks are not expected to be more severe than those caused by methicillin-susceptible *S. aureus* [13]. The enterotoxigenic potential of CoNS has not been well-established, even though studies have evidenced the presence of enterotoxin-coding genes and the production of enterotoxins in CoNS [14].

Studies on the epidemiology and genetic diversity of methicillin-resistant staphylococci in the dairy production chain are important to promote food safety. Such studies contribute to a better understanding of MRS and to the development of measures to control and prevent their dissemination. This study therefore focused on isolating MRS at dairy farms and in the production of artisanal unpasteurized cheese, and aimed to characterize them in terms of their antimicrobial susceptibility, gene sequencing, and genetic diversity, thereby contributing to the body of knowledge about the epidemiology of MRS.

## 2. Results

The 391 isolates yielded 16 *mecA* positive isolates (MRS), but none of them were identified as *S. aureus* species. Three MRS isolates were obtained from cheese handler’s hands and four from artisanal unpasteurized cheese produced at farm B. In addition, five MRS isolates were obtained from cheese handler’s hands and four from artisanal unpasteurized cheese produced at farm C. And from 16 *mecA* positive isolates (MRS), none isolate had enterotoxin genes *(sea, seb, sec, sed, see, seg, seh, sei, tst, eta, pvl,* and *hlg*).

These MRS isolates were sensitive to rifampicin, vancomycin, clindamycin, gentamicin, and tetracycline; nonetheless they were resistant to several antimicrobials, such as oxacillin, penicillin, and cefepime. Isolates obtained from farm C were also resistant to ciprofloxacin (Table 1).

**Figure 1:**
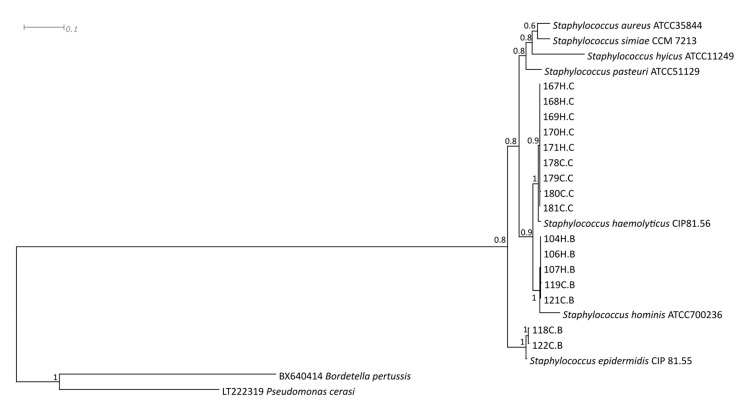
Bayesian phylogenetic tree of the *rpoB, gap* and *tuf* genes concatenated sequences from MRS isolates identified at two non-technified dairy farms that produce artisanal unpasteurized cheese in Brazil. H.B: isolates from cheese handler’s hands at farm B; C.B: isolates from artisanal unpasteurized cheese produced on farm B; H.C: isolates from cheese handler’s hands at farm C; and C.C: isolates from artisanal unpasteurized cheese produced on farm C.

The MRS isolates found in this study were identified as *S. haemolyticus, S. hominis,* and *S. epidermidis* by phylogenetic analysis of the *rpoB*, *gap*, and *tuf* genes (Table 2; Figure 1).

**Figure 2:**
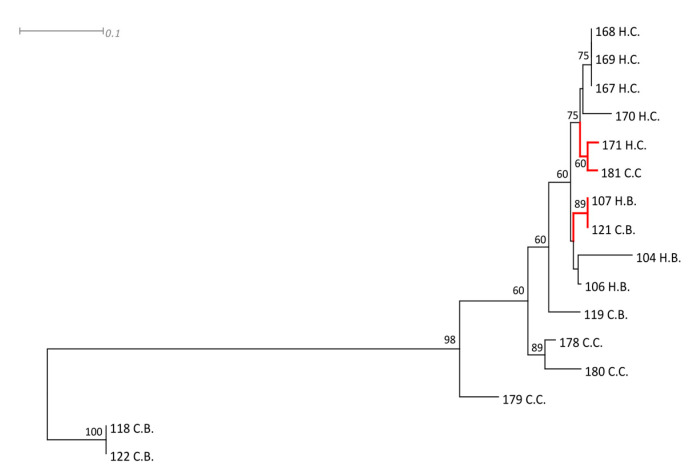
Neighbor-joining dendrogram of MRS isolates from two non-technified dairy farms that produce artisanal unpasteurized cheese in Brazil. The clusters in red marker indicate genetic similarities between strains obtained from cheese handler’s hands and from cheese ready for consumption. H.B: isolates from cheese handler’s hands at farm B; C.B: isolates from artisanal unpasteurized cheese produced at farm B; H.C: isolates from cheese handler’s hands at farm C; and C.C: isolates from artisanal unpasteurized cheese produced at farm C.

The MRS strains were clustered according to their species and genetic similarities, related to common sources of contamination, through RAPD analysis. Two main clusters were formed, one composed of *S. haemolyticus* and *S. hominis* strains and the other by the two isolates of *S. epidermidis* (Figure 2). Isolates from cheese handlers’ hands and artisanal unpasteurized cheese from both Farm B (107H.B. and 121 C.B.) and Farm C (171 H.C. and 181 H.C.) showed high genetic similarity between them, which indicates cross contamination during cheese production. The other isolates were grouped according to their source and the farm from which they were obtained.

## 3. Discussion

The detection of MRS isolates at dairy farms may be attributed to the presence of animals with clinical mastitis and to the improper use of antimicrobials in their treatment. It is known that methicillin-resistant staphylococci can occur due to the use of intra-mammary antibiotics to treat mastitis [15], which may explain the high values found mainly at Farm C. The transmission of antimicrobial resistant genes from dairy cows to farm workers and residents has been previously reported [16].

The antimicrobial resistance of *Staphylococcus* spp. is a serious problem because antimicrobial resistance genes are transferred to distinct strains and species through the exchange of genetic material. Antimicrobial-resistant strains are detected in animals and can be transmitted to food containing animal products [17]. MRSA strains have been found in milk from cows infected with mastitis and unpasteurized cheese, underscoring the possibility of transmission from cows to dairy products [18,19], even though MRSA had not been detected in this study. Nonetheless, the coagulase-negative methicillin-resistant staphylococci has equal importance, as once they are opportunistic pathogens, with the ability to cause mastitis, they become a source of resistant zoonotic infection and reservoir of antimicrobial resistance genes in dairy farms [3]. CoNS are widely reported in dairy farms and have been reported in milk [20], mastitis [21], biofilms [22], milkers’ hands and farm environment [23]. The dissemination of methicillin-resistant staphylococci in cows, humans, and manure has been proven, indicating the possible transmission of methicillin-resistance to humans in contact with cows and manure [24].

None of the 16 MRS isolates had enterotoxin genes and this can be justified by the fact that they are CoNS and these genes are not so common in these isolates. A study in coagulase-negative *Staphylococcus* spp. from buffalo milk and the milking environment in Brazil showed only two strains positive for the *see* and *eta* toxin genes [25]. Also, coagulase-negative *Staphylococcus* spp. of subclinical mastitis in sheep also showed only the *sec* gene related to the production of enterotoxins [26].

The isolates obtained in this study were resistant to important antimicrobials used in human medicine, as reported in England and Wales, where coagulase-negative, methicillin-resistant staphylococci strains encountered in dairy farms were also resistant to fusidic acid, tetracycline, and clindamycin [3]. In addition, resistance to methicillin, oxacillin, penicillin, ampicillin, cephalothin, kanamycin, gentamicin, and tetracycline was reported in MRS obtained from samples of bovine mastitis in Korea [27].

At farm B, the person who did the milking was also involved in unpasteurized cheese production. Our findings suggest that milk and dairy products represent a potential risk of multidrug resistant infections due to poor hygiene practices during the production of artisanal unpasteurized cheese. Moreover, we emphasize the need to adopt more restrictive hygiene strategies in the dairy production chain in order to promote food safety. High quality safe unpasteurized milk is directly linked to healthy animals and meticulous attention to good hygiene practices. Proper farmer training and support programs are very important to help dairy producers understand the various risks in milk production and the measures needed to mitigate such risks. Actions to reduce the possibility of contamination of unpasteurized milk and cheese are essential and must be practiced daily to ensure consistent product safety [28].

Several studies have described MRS or MRSA in foods [29,30,31] and in hospital patients [32]. However, no published data were found evidencing the transmission of MRS from food handlers to food. The present study reports this kind of cross contamination by demonstrating the high genetic similarity between strains from artisanal unpasteurized cheese and human hands. It should also be kept in mind that, at each farm, milking and cheese processing were performed by the same person. This type of contamination can easily be avoided by adopting good milking and cheese production practices. Therefore, farmers should receive training about good hygiene practices in milking and in the production of unpasteurized cheese, as well as about the proper treatment of infected cows. In addition, the routine inspection of artisanal unpasteurized cheeses could be useful in order to control widespread MRS and reduce risks to public health. Besides *S. aureus* being considered the most important foodborne pathogen, other CoNS strains have been detected carrying enterotoxin-coding genes and the production of enterotoxins has been proven [14] although they are not so common. In addition, methicillin-resistant staphylococci can be a source of zoonotic infections and a reservoir of antimicrobial resistant genes [3], contributing to widespread antimicrobial resistance and requiring urgent control.

In conclusion, this study identified the major role of people working in milking and artisanal unpasteurized cheese production in the dissemination of methicillin-resistant *Staphylococcus* species through improper product handling procedures and the consequent risks to public health.

## 4. Material and Methods

### 4.1. Staphylococcus spp. Sampling, Isolation and Identification

In 2014, samples were collected of bovine feces, swab samples from milkmen’s and cheese handlers hands, from milking buckets, samples of unpasteurized artisanal cheese, whey, water, cheese processing surfaces, sieves, trays, molds, and skimmers at five non-technified dairy farms (A, B, C, D, E) in the northeastern region of the state of São Paulo, Brazil [33]. These farms used manual milking and produced artisanal unpasteurized cheese. The person that did the milking at each farm was also the one directly involved in cheesemaking. At least 17 samples (one of each type of sample and five of bovine feces) were collected at each farm, making a total of 106 samples, 22 from farm A, 23 from B, 21 from C, 21 from D, and 19 from E. All these samples were put up in peptone water 0.1% and enriched in Brain Heat Infusion (BHI) broth with NaCl 6%.

*Staphylococcus* spp. was isolated as described by [34]. Three to five suggestive colonies of *Staphylococcus* spp. were collected from each Petri dish containing mannitol salt agar (MSA) with oxacillin 2 μg.mL^−1^. Suggestive colonies of *S. aureus* were those colored in yellow with yellow zones while coagulase-negative *Staphylococcus* were small pink or red colonies with no color change in the medium. A set of 391 isolates were obtained and transferred to tubes containing BHI broth.

DNA was extracted as described by [35] and multiplex PCR was used to confirm the presence of *Staphylococcus* spp. [36], *S. aureus* [37] and *mecA* gene presence [38].

### 4.2. Detection of Enterotoxin Genes

MRS isolates were tested for the presence of toxin encoding genes (*sea, seb, sec, sed, see, seg, seh, sei, tst, eta, pvl,* and *hlg* [39].

### 4.3. Antimicrobial Susceptibility Test of MRS Isolates

MRS isolates were subjected to an in vitro sensitivity test using the disk diffusion method [40]. The antimicrobials tested were cefepime (30 μg), ciprofloxacin (5 μg), chloramphenicol (30 μg), clindamycin (2 μg), erythromycin (15 μg), gentamicin (10 μg), oxacillin (1 μg), penicillin G (10 μm), rifampicin (30 μg), sulfatrim (25 μg), tetracycline (30 μg), and vancomycin (30 μg) [41].

### 4.4. Sequencing

DNA was extracted from MRS isolates [42]. The identification of MRS species was performed by sequencing of *rpob*, *gap,* and *tuf* conserved genes using the primers proposed by [43,44] and [45], respectively. Reactions were prepared with 1× buffer (20 mM Tris-HCl pH 8.4, 50 mM KCl), 2 mM MgCl_2_, 0.2 mM dNTPs, 0.5 U of Platinum^®^ Taq DNA polymerase (Invitrogen), 4 pmol of primers and 60 ng of genomic DNA and nuclease free water up to 20 µL. Amplified PCR products were subjected to sequencing by using the BigDye^®^ Terminator v3.1 kit (Thermo Fisher Scientific, Waltham, MA, USA) and capillary electrophoresis was performed in an ABI3130 sequencer (Applied Biosystems, Foster City, CA, USA).

Quality trimming of reads was achieved using the Phred/Phrap/Consed package, considering Phred base quality equal to or higher than 20 [46,47,48]. Sequences of the three genes and those available in the GenBank were aligned using Multiple Sequence Comparison by Log-Expectation (MUSCLE) [49]. *Bordetella pertussis* (Accession number BX640414.1) and *Pseudomonas* sp. (LT222319.1) were used as outgroups. Aligned sequences of the three genes were concatenated and the best fit model for phylogenetic analysis was selected, based on the Akaike information criterion (AIC) [50]. Bayesian analyses were performed using MrBayes v. 3.2.3 software [51] with the number of substitution six and rates gamma. Four independent runs were carried out with 5,000,000 generations, with sampling performed at intervals of 100 generations. At the end of the analyses, with a standard deviation less than 0.01, the initial 25% of the trees was discarded as burn-in. The resulting phylogram was edited graphically using the software Dendroscope 3 [52].

### 4.5. RAPD Markers

MRS genetic diversity was analyzed using RAPD (Random Amplified Polymorphic DNA) molecular markers. One hundred RAPD primers were tested (Operon Technologies, CA, USA) in order to select the most informative and polymorphic ones. Twelve primers were selected: OPA4, OPA13, OPA18, OPG14, OPI7, OPM5, OPM12, OPP19, OPQ1, OPQ18, OPR2, and OPR12. The amplification reaction of fragments was performed with 1× buffer (20 mM Tris-HCl pH 8.4, 50 mM KCl), 2.5 mM MgCl 2, 0.2 mM dNTPs, 1.0 U Taq DNA polymerase (Fermentas, Thermo Fisher Scientific), 5 pmol of primer, 60 ng of genomic DNA and pure sterile water to 20 μL. Amplification was performed in a Eppendorf Nexus thermal cycler programmed to cycle at 92 °C for 3 min, 35 cycles at 92 °C for 60 s, 36 °C for one min and 45 s, 72 °C for 1 min and 45 s, and at 72 °C for 10 min. Amplification products were separated using 1.5% agarose gel electrophoresis stained with ethidium bromide (0.5μg/mL).

Banding patterns were analyzed to obtain a binary matrix, which was used to generate a distance matrix [53] and a neighbor-joining cluster with 1000 bootstraps [54,55] using PAUP 4.0b10 software [56]. The dendrogram obtained was graphically edited using the software Dendroscope 3 [52].

## Figures and Tables

**Table 1:** Resistance of MRS strains to seven different antimicrobials observed at two non-technified dairy farms that produce artisanal unpasteurized cheese in Brazil.

Farm	Sample	IsolateS (N)	Antimicrobial Drug						
			CPM	CLO	ERI	PEN	OXA	SUT	CIP
B	Cheese handlers‘ hands	3	3 (100)	0	1 (33,33)	3 (100)	3 (100)	3 (100)	0
	Artisanal cheese	4	2 (50)	0	0	4 (100)	4 (100)	2 (50)	0
C	Cheese handlers‘ hands	5	2 (40)	0	0	5 (100)	5 (100)	5 (100)	5 (100)
	Artisanal cheese	4	0	3 (75)	1 (25)	4 (100)	4 (100)	3 (75)	4 (100)

CPM, Cefepime; CLO, Chloramphenicol; ERI, Erythromycin; PEN, Penicillin; OXA, Oxacillin, SUT, Sulfatrim; CIP, Ciprofloxacin.

**Table 2:** Accession numbers of MRS strains from two non-technified dairy farms that produce artisanal unpasteurized cheese in Brazil, and Staphylococcus spp. strains from the GenBank used in the phylogenetic analysis.

*Staphylococcus* Species	Strain	*rpoB*	*gap*	*tuf*
*Staphylococcus aureus*	ATCC 35844	AF325894	HM352968	HM352930
*Staphylococcus epidermidis*	CIP 81.55	EU659944	EU659906	EU652794
*Staphylococcus haemolyticus*	CIP81.56	EU659950	EU659920	EU652800
*Staphylococcus hominis*	ATCC700236	MF679108	HM352973	HM352925
*Staphylococcus hyicus*	ATCC 11249	AF325876	FJ578002	CP008747
*Staphylococcus pasteuri*	ATCC 51129	EU659961	HM352972	HM352929
*Staphylococcus simiae*	CCM 7213	EU888127	HM352970	HM352931
*Staphylococcus hominis*	104H.B	MT832255	MT832271	MT832239
*Staphylococcus hominis*	106H.B	MT832256	MT832272	MT832240
*Staphylococcus hominis*	107H.B	MT832257	MT832273	MT832241
*Staphylococcus epidermidis*	118C.B	MT832258	MT832274	MT832242
*Staphylococcus hominis*	119C.B	MT832259	MT832275	MT832243
*Staphylococcus hominis*	121C.B	MT832260	MT832276	MT832244
*Staphylococcus epidermidis*	122C.B	MT832261	MT832277	MT832245
*Staphylococcus haemolyticus*	167H.C	MT832262	MT832278	MT832246
*Staphylococcus haemolyticus*	168H.C	MT832263	MT832279	MT832247
*Staphylococcus haemolyticus*	169H.C	MT832264	MT832280	MT832248
*Staphylococcus haemolyticus*	170H.C	MT832265	MT832281	MT832249
*Staphylococcus haemolyticus*	171H.C	MT832266	MT832282	MT832250
*Staphylococcus haemolyticus*	178C.C	MT832267	MT832283	MT832251
*Staphylococcus haemolyticus*	179C.C	MT832268	MT832284	MT832252
*Staphylococcus haemolyticus*	180C.C	MT832269	MT832285	MT832253
*Staphylococcus haemolyticus*	181C.C	MT832270	MT832286	MT832254
